# The Mixed Effect of China’s New Health Care Reform on Health Insurance Coverage and the Efficiency of Health Service Utilisation: A Longitudinal Approach

**DOI:** 10.3390/ijerph17051782

**Published:** 2020-03-09

**Authors:** Jiaqi Chen, Song Xu, Jing Gao

**Affiliations:** 1 School of Business, Jiangnan University, 1800 Lihu Ave, Binhu District, Wuxi 214122, China; jiaqichen@jiangnan.edu.cn (J.C.); gj_jessica1982@hotmail.com (J.G.); 2 School of Medicine, Jiangnan University, 1800 Lihu Ave, Binhu District, Wuxi 214122, China

**Keywords:** health care reform, health insurance coverage, health service utilisation, health resource allocation

## Abstract

In 2009, China launched a new health care reform as it endeavoured to develop a tiered system of disease diagnosis and treatment to promote the integration of medical resources. This was important for improving service capacity and building medical alliances that would eventually lead to improved health service utilisation efficiency. However, while the 2009 reform aimed to provide universal health insurance coverage to all citizens, its overall effect on health service utilisation efficiency remains unclear. We aimed to examine the new health care reform’s mixed effect by applying a longitudinal study using China Health and Nutrition Survey (CHNS) data and the difference-in-difference (DID) method to estimate the health reform’s impact on health insurance coverage rate. Then, we studied whether the increase in health insurance coverage rate affected health service utilisation efficiency in China. Our results showed that the increase in insurance coverage rate has indeed made expensive medical services available to low-income individuals. However, it also increased the likelihood of use of hospitals rather than primary care facilities, since there is more insurance cover for outpatient visits, which has led to an increased demand for quality services. This effect has generated a negative impact on health care utilisation which directly pertains to systemic inefficiency. This study thus indicates that China’s latest health reform requires further policies to improve its overall efficiency.

## 1. Introduction

Over the past 70 years, China has made significant progress in providing efficient and affordable health care services to a large portion of its population. Notably, the country has implemented more than five large health care reform waves since the 1950s to meet the rapid increase in demand for an efficient medical service system. In 2009, China initiated a new and thorough health care reform aiming to provide universal health coverage by 2020 with equal access to basic health care at reasonable quality. In an independent assessment, Yip and colleagues found that from 2008 to 2017, the health expenditures spent by the Chinese government on health care quadrupled from 359 billion yuan to 1.52 trillion yuan [1].

Under the 2009 health care reform’s framework, the Chinese government intended to deliver additional reforms to overhaul its hospital-centric, treatment-based delivery system [2]. In fact, strengthening primary health care systems (PHC) and diverting patients with common diseases from hospital-based care to community-based care is one of the top priorities of health care reform [3,4]. The government also introduced a policy to encourage a tiered diagnosis and medical treatment system meant to optimise medical resources and promote medical service integrity while alleviating medical costs [5]. The ultimate goal of this reform, then, is to sink medical resources from tertiary hospitals into PHC systems [6].

Balancing health service resources under this new reform is therefore a critical concern researchers and authorities face due to the limited capacity among various medical units, which from the supply side can determine access to health care. Previous research has reported a positive relationship between health resources and accessibility to health care systems [7,8,9,10]. Meanwhile, other researchers have investigated the overcrowding problems in major Chinese hospitals and evaluated the impacts of service interaction and blocking on patient flow [11]. However, despite the many efforts that China’s 2009 health care reform has made, inefficient medical service systems and patients’ valuing of service quality are major obstacles to diverting patients with common diseases to community-based medical centres [12,13,14,15]. This is because patients care more about the quality of the provided health services rather than fees when the total cost is affordable [16,17,18]. There are also problems reported in the quality of care and control of health expenditures [19,20,21,22].

As many public policies endeavour to optimise scarce resources, these different policies have had mixed effects. Although health care services are not free to all its citizens, the Chinese government has made efforts to increase public health insurance coverage. Recent studies have reported that Chinese citizens’ medical expenditures and out-of-pocket (OOP) payments, especially for catastrophic illnesses, have indeed decreased in volume [1]. However, the alleviation of medical costs has led to more patients going to upper-level hospitals for both minor and serious illnesses, not to community health service institutions, despite the fact that the latter offers both cheaper and timelier medical services compared to major hospitals. Additionally, the general perception of patients in China is that community health care services lack well-trained doctors, medicines, and equipment. Therefore, it is important to examine the effects of Chinese health care reforms after 2009 and evaluate health care resource utilisation. Audibert et al. (2013) studied the cross-effect of health care reforms and the efficiency of township hospitals, finding that the introduction of the 2009 health care reform had a negative effect on the evolution of efficiency among township hospitals [23]. Based on these findings, this paper aims to study the mixed effects of the two policies: whether the introduction of the universal health insurance coverage policy has a negative effect on the policy that encourages a tiered health service system to improve health service utilisation efficiency.

So far, many studies have investigated the influence of China’s health care reforms. However, very few have evaluated the 2009 health care reform in terms of health insurance coverage, and more importantly, the corresponding effect of the increased insurance coverage rate on health service utilisation efficiency. Our study thus had three objectives: first, to determine whether China’s 2009 health care reform has achieved universal insurance coverage for all citizens; second, to evaluate the corresponding effect of the increased insurance coverage rate on health service utilisation efficiency; and third, to detect the probability of individuals who are beneficiaries of the new health care reform overcrowding tertiary hospitals. We used longitudinal data from the China Health and Nutrition Survey (CHNS) and the difference-in-differences (DID) method to measure and analyse the proposed research question. This paper extends the existing literature to investigate the causal relationships between health care reforms, health insurance coverage rates, and individuals’ decisions to visit upper-level hospitals through an econometric analysis. We further contribute to the literature by proposing these research questions and addressing the promotion of China’s tiered medical system and alleviated medical expenditures to achieve overall health service utilisation efficiency.

The remainder of this paper is organised as follows. Section 2 briefly reviews the history of China’s health insurance reforms, especially focusing on the most recent 2009 reform. Section 3 presents our data source and methodology. The main results are discussed in Section 4; Section 5 introduces the main findings. Section 6 concludes the study.

## 2. A Review of China’s Health Insurance Reforms

For decades, the Chinese government has aimed to provide adequate health insurance to the majority of its citizens in a cost-effective and efficient manner. In the early 1950s, China established its social medical insurance system. However, this system was designed specifically for urban workers. Afterwards, though, several waves of health care reforms took place to expand the country’s insurance program [24]. At the end of 1998, the Chinese government established the insurance reform known as the Urban Employees’ Basic Medical Insurance (UEBMI). At the time, it was a new social health insurance system for all urban workers, including state-owned enterprise (SOE) employees, government-run facility personnel, and private company workers. The introduction of the UEBMI system was a primary policy governing health insurance provision [25], with the Chinese government implementing three further waves of health care reform. Because these large-scale reforms could affect more than a billion people, the health reforms were developed with changing priorities. These three post-UEBMI reform waves are known as the New Rural Cooperative Medical Scheme (NRCMS) for the rural population; the Urban Resident Basic Medical Insurance (URBMI) system, which was aimed at non-employed residents living in urban areas, including children, the elderly, and people who are disabled or poor; and the most recent 2009 health care reform, which aimed to provide universal health coverage [26,27].

This latest health care reform has been regarded as the most ambitious in history. Over the course of the reform, its implementation was backed by the strongest support, with the Chinese government quadrupling its financial support for the health reform from 2008 to 2017 [1]. Moreover, this health care reform’s leading group was chaired by the vice premier for policy coordination. This reform has even attracted international attention, since it can provide valuable implications for other countries around the world [28].

The 2009 reform was divided into two phases. The first was from 2009 to 2012, with the priority to expand social health insurance coverage for all people and strengthen China’s PHC system. The second phase started in 2013. For this stage, the reform focused more on reducing inefficiencies in the health care system, with relevant policies including encouraging systemic reforms for public hospitals and establishing medical alliances [29].

China’s health care reforms have received a significant amount of attention from researchers, with evaluations of the 2009 health care reform’s impact especially being mainly focused on the following aspects. One stream of literature discusses the reform’s effect on health care expenditures. Yang et al. (2016) found that the reform significantly reduced hospitalisation expenses in pilot hospitals [30]. Liu et al. (2002), however, suggested that in spite of all of China’s reforms up to this study’s publication, people with poor socioeconomic status were still disadvantaged in accessing expensive and advanced health services [25]. Their findings are further supported by Atelia et al. (2015), who argued that OOP expenses decrease only for individuals with high income and who are generally in good health [31]. Additionally, there are studies that focus on health service utilisation efficiency. For example, Zhang et al. (2011) argued that Chinese PHC service centres are unable to attract patients due to a lack of operational efficiency [32]. Yip et al. (2012) applied a comprehensive method to evaluating the 2009 health care reform, and their findings suggested that the transformation of the universal insurance coverage rate into cost-effective services is difficult. This is due to inefficiencies, the poor quality of the health services provided, and scarcity issues that require further investigation [33].

While existing initiatives in the 2009 health system reform show significant progress in many aspects [34,35], there is little evidence of the effect of true insurance coverage and direct trends in access to health services. Moreover, few studies have focused on the reform’s mixed effect of increased insurance coverage on health care utilisation efficiency, especially the negative impact of one of its policies over another. Performing an econometric analysis with regard to these indicators would help researchers and policy makers better understand the real achievements of this reform, and any remaining challenges further ahead. These indicators are in fact the essential goals of China’s 2009 health system reform. Therefore, two questions need to be explored: what is the effect of the public health insurance coverage expansion, and how does this affect health service utilisation efficiency? This paper strives to answer these two questions in regard to China’s 2009 health care reform.

## 3. Methods

### 3.1. Data

We used CHNS data from 1989 to 2015 to conduct our analysis. In the CHNS, a multistage cluster sampling is used to randomly draw individuals from China’s nine provinces. The data thus contains questions for individuals on socioeconomic status and health outcomes: for example, regarding health service utilisation, insurance coverage, health facilities, etc. [36]. In terms of health insurance coverage, the survey asks the following questions: (1) “Do you have medical insurance?” (2) “If yes, which type of medical insurance do you have?” The insurance classification varies from public to commercial health insurance. As for the health service utilisation efficiency, the survey asks: (1) “Did you seek formal medical care in the last four weeks?” (2) “Where did you see a doctor?” (3) “How much is your treatment cost?” (4) “What is the percentage that is covered by insurance?” (5) “How much is the additional yuan spent?”.

For our main analysis, we evaluated the effect of the 2009 health care reform both before and after its announcement. Therefore, following a common selection rule of the sample period in the literature (see for example, He and Nolen [37]), we assessed the 2006, 2009, 2011, and 2015 reform waves. We also used the DID estimator to examine the causal effect of the latest health care reform on rates of insurance coverage. We applied the DID approach to alleviate potential endogenous problems when evaluating the causal relationship between health insurance coverage rate and overcrowding in upper-level hospitals as well. The 2006 wave survey served as our pre-treatment (pre-first stage reform) wave and the 2011 survey as our post-treatment (post-second stage reform) wave since it captures the immediate effect after the application of the 2009 health care reform. We also applied multiple placebo tests for further analysis. Then, we analysed both the 2011 and 2015 waves in our post-treatment analysis. We included 56,873 individuals, of which 64.24% lived in rural areas and 35.76% in urban areas when the survey was taken. To note, urban and rural living status is based on where the participating individuals lived during the survey period rather than their actual hukou residences.

### 3.2. Methods and Research Design

The use of the DID approach required us to observe a treatment and control group over at least two time periods: for instance, before and after the 2009 health care reform [38,39]. Since the 2009 health care reform was intended to provide basic insurance to all Chinese citizens, especially the unemployed, these citizens were our treatment group, as they were not covered in the 2006 wave, but indeed, in the 2011 and 2015 waves. For the control group, we used SOE employees, since they were covered in the insurance plans in both periods—that is, the 2009 health care reform did not affect this group. According to the theory and in the context of our study, the DID approach assumes that in the absence of the health care reform, the time trend should have been the same for both the treated and control groups. We relaxed this condition a little bit by adding individual controls. However, the assumption still holds, given that our sample was a subset of the labour force. However, we used data from both urban and rural areas to examine how likely it is that our assumption is correct. Given this set-up, we estimated the following:

(1) $$\begin{array}{c} E\left[Y_{i}|T=Unemployed,P=after\right]-E\left[Y_{i}|T=Unemployed,P=before\right]\\ -E\left[Y_{i}|T=SOEemployed,P=after\right]-E\left[Y_{i}|T=SOEemployed,P=before\right] \end{array}$$

The difference-in-differences regression model was also applied in the following equation:

(2) Y(i,t)=δ(t)+α∗D(i,t)+η(i)+υ(i,t)

where δ(t) is the time specific component, η(i) is the individual component, υ(i,t) the is control variable, and α represents the impact of the treatment. Additionally, we followed this equation:

(3) $$Y_{i,t}=\beta_{0}+\beta_{1}T_{i}+\beta_{2}P_{t}+\beta_{3}T_{i}*P_{t}+\upsilon \left(i,t\right)$$

where $Y_{i,t}$ measures the take up of insurance, the utilisation of health service, and out-of-pocket expenditure. $T_{i}$ took on the value one if the individual never worked during the survey period, and 0 otherwise. $P_{t}$ is the dummy variable that takes the value 1 if the time is after the reform, and 0 otherwise. In particular, we are interested in the value of $\beta_{3}$, since it is a difference in difference coefficient, which is actually measured as follows:

(4) $$\bar{\beta_{3}}=\left(\bar{y_{unemployed,2}}-\bar{y_{unemployed,1}}\right)-\left(\bar{y_{u,2}}-\bar{y_{u,1}}\right)$$

Since the unemployed individuals and SOE employees were not randomly assigned, we wanted to control for possible differences in the two groups’ characteristics. As a result, we applied individual covariates, which means the time trend in the outcome variable would have been the same for both groups. We therefore estimated the following model:

(5) $$Y_{i,t}=\beta_{0}+\beta_{1}T_{i}+\beta_{2}P_{t}+\beta_{3}T_{i}*P_{t}+\theta I_{i,t}+\upsilon \left(i,t\right)$$

where $I_{i,t}$ denotes a individual characteristics vector. In this model, the coefficient $\beta_{3}$ is the estimate of interest.

While urban areas are not ideal comparisons for what might be happening in rural areas, if an unemployed individual has a directly positive effect on health outcomes independent of the 2009 health care reform policy, then we should see a trend in urban areas when we compare unemployed individuals to SOE employees. Therefore, we used the following model to capture the estimate from the equation below:

(6) $$Y_{i,t}=\beta_{0}+\beta_{1}T_{i}+\beta_{2}P_{t}+\beta_{3}T_{i}*P_{i}+\delta_{0}U_{i}+\delta_{1}U_{i}*T_{i}+\delta_{2}U_{i}*P_{t}+\delta_{3}U_{i}*T_{i}*P_{t}+\upsilon \left(i,t\right)$$

where $U_{i}$ is a dummy variable equal to 1 if the observation is from a urban area and zero otherwise.

Next, we studied the effect of the 2009 health care reform and health service utilisation efficiency in terms of the delivery of tertiary hospitals’ health care services. It is worth noting that there is no health care gatekeeping system in China, meaning that patients can choose whether they go to tertiary hospitals or PHC centres. We argue that while the latest health insurance reform intended to increase health insurance coverage to produce commensurate benefits for all Chinese citizens, it also caused city hospitals to overcrowd with patients seeking cures for common diseases, leaving limited resources for catastrophic illnesses. This might be due to the increase in insurance coverage alleviating health expenditure burdens, making city hospitals affordable and cost-effective. We thus used the following econometric specification:

(7) $$UpperHospital_{i,t}=\beta_{0}+\beta_{1}Ins+\beta_{2}OOP+\beta_{3}Ins*OOP+\beta_{4}F_{i,t}+\upsilon \left(i,t\right)$$

where $UpperHospital_{i,t}$ is the dependent variable, measuring whether or not the individual chooses to visit city upper-level hospitals; Ins is the cost covered by health insurance; OOP is the out-of-pocket health expenditure. The interaction variable measures the impact of increase in costs covered by health insurance on alleviating health expenditure and on the take up of city hospital facilities. $F_{i,t}$ are control variables related to this analysis which include age, district, unemployment condition, and gender factors. Since the above equation may subject to endogenous problems, we again apply the DID approach to alleviate potential endogeneity as a robustness check.

## 4. Main Results

### 4.1. The Effect of Deep Health Insurance Reform on Health Insurance Coverage

**Figure 1:**
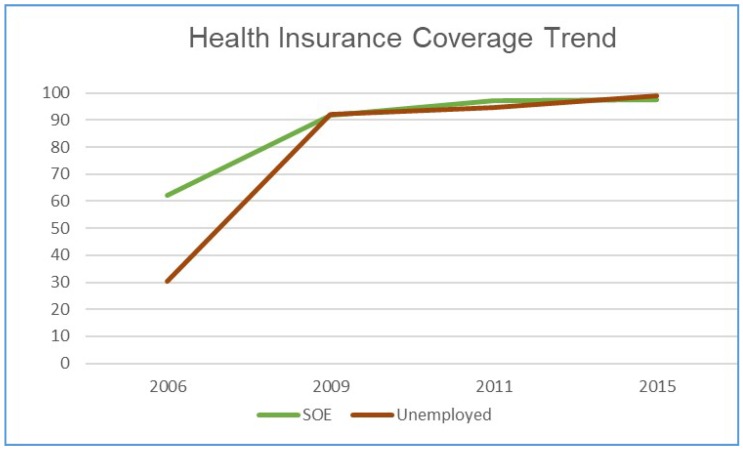
Health insurance usage by type and wave.

Figure 1 summarises the health coverage rate for the two sample groups in our sample. The insurance coverage rate remained low at 30.54% for the unemployed in 2006, while for SOE employees, the rate was 62.15%. These rates increased significantly after the health reform in 2009, with more than 97% of the sample individuals having basic medical insurance coverage.

The public health insurance coverage rate also expanded rapidly in both urban and rural China. Table 1 reports the health insurance rise across urban and rural districts and among the four time periods. Overall coverage measures the rate of respondents who reported having at least one health issue covered by insurance. Free health insurance, meanwhile, was established to cover civil servants’ medical expenses in the early 1950s, including inpatient and outpatient services. The usage rate of all types of insurance increased from 49.51% in 2006 to 96.09% in 2015 for urban residents and from 47.63% to 97.77% for rural residents.

From the more detailed descriptive statistics in Table 1, we studied whether the launch and implementation of the major public health insurance schemes in each time period played significant roles in rapid changes in insurance coverage rate. Indeed, the insurance coverage rate in urban system (UEBMI and URBMI) rose from 13.29% in 2006 to 73.14% in 2015. The UEBMI covered 13.29% of the urban sample in 2006, but increased three times by 2009 to 39.7%; meanwhile, the URBMI covered 17.74% only two years after its introduction in 2007. The coverage rates were high in rural areas due to the popularity of the NRCMS program, with all at more than 70%. There was also a large percentage of individuals who were living in urban areas at the time of the survey but who were covered by NRCMS, and conversely, urban individuals living in rural areas who had urban insurance. This partially reflects the migration of the rural population to urban areas, since the number of NRCMS holders is much higher in urban areas. The coverage of free government insurance also rapidly dropped from 10.77% in 2006 to 4.49% in urban places, but remained around 2% in rural areas.

Next, we applied a detailed analysis on the effects of the health care reform. Table 2 presents the definitions of the key variables used in this study, whereas Table 3 and Table 4 show the key variables’ descriptive statistics. Specifically, Table 3 shows trends in health service utilisation and people’s choices regarding tertiary hospitals, with health service utilisation increasing significantly after the 2009 health care reform. However, gaps also remain between urban and rural residents in health service utilisation. A further exploration of people’s choices regarding tertiary hospitals shows that the proportion of people who prefer tertiary hospitals increased after the reform. Still, the percentage does not vary between urban and rural residents.

We present a broader picture in Table 4. The 2006 sample had 9661 individuals, and the 2015 sample had 12,944. The insurance coverage rate was at an average of 84.9%, and total OOP medical expenditures were at an average of 958 yuan. As depicted in Table 4, we also observed an increase in insurance coverage and OOP expenditures. The average real total OOP expenditures increased by 84% from 2006 to 2015, from 699 to 1284 yuan. The remaining variables in Table 4 served as our control variables: 52.6% of the sample individuals were female; 5.2% were unemployed; 37.7% were from urban areas; the average age was 56; and the average length of education the individuals received was eight years.

We then examined the trends in insurance coverage rate after the 2009 universal health care reform. Table 5 presents the simple DID results. Column (1) describes the general results. We report that insurance coverage significantly increased among unemployed individuals, while there was no significant change among SOE employees. The triple difference reported in column (3) is 27.8, which is significant at the 1% confidence level. This suggest that if the difference in health insurance coverage between unemployed individuals and SOE employees was increasing even before the introduction of the 2009 health care reform, the increase in urban areas was more than the rate reported in the trend from rural areas, showing that the provision of the new health reform had a large and significant effect. There is strong evidence that the new health care reform increased the coverage rate. We pushed this assertion further by controlling for individual covariates to see if it still held true, with the results reported in column (2). We found that after controlling for individual covariates, the DID estimation captured a similar trend.

### 4.2. The Effect of Increase in Health Insurance Coverage and the Efficiency of Health Service Utilisation

We continued on to study the 2009 health care reform’s effect on health insurance coverage and its corresponding impact on the utilisation of health services and tertiary hospitals. We report the regression results of the logistic models in Table 6. The use of tertiary hospitals is the recent event and the comparative group is made up of people who reported using a PHC service when ill in the previous four weeks. We discovered that the increase in general health insurance coverage rate increased the total percentage of costs covered by insurance. The significant negative crossed coefficient further indicates that the use of tertiary hospitals was associated with a higher OOP and with higher percentage covered by health insurance.

## 5. Discussions

The Chinese government has been diligently reforming its health care system since 2009. This study evaluated the proportion of public health insurance usage, the trends in health service utilisation, the mixed effect of these two trends, and further, people’s decisions to visit tertiary hospitals. We have the following findings. First, the 2009 health care reform shows encouraging results in trends in the proportion of insurance coverage and health service utilisation. Compared to Hou and Zhang’s (2017) findings, our results show a consistent increase in trend following the 2009 health care reform [27]. Moreover, the DID results extend the findings of He et al. (2019) that the latest health care reform further heightened the insurance coverage rate, with substantial coverage to unemployed individuals especially [37]. Second, further analysis indicates that the increase in health insurance coverage increased the total cost covered by insurance, which produced commensurate benefits for individuals in less OOP expenditures. This eventually led to more individuals crowding into tertiary city hospitals.

Furthermore, to alleviate potential endogeneity problems, we applied the DID estimation as a robustness check. Table 7 reports the robustness test results of the above regression models. Column (1) features the simple DID results, while column (2) outlines the DID results with covariates. We observed that the 2009 health care reform increased the use of health care services. The DID estimate coefficient (0.0148 at 1% significance level) further shows that due to this health care reform, there was a significant increase in urban residents’ preference for tertiary hospitals.

In addition, since the treatment group was composed of unemployed citizens, their health status, health literacy, and living behaviours could be different. Therefore, we applied a robustness test by using non-SOE employees as the control group, as the responsiveness to health insurance coverage could be more similar between these two groups. Table 8 reports the 2009 health care reform’s effect on insurance coverage by using non-SOE employees as the control group. Our results are robust, and the statistically significant positive cross coefficients suggest that the 2009 health care reform had a positive impact on insurance coverage rate, especially for unemployed citizens.

## 6. Conclusions

This paper evaluated the impact of China’s latest health care reform from 2009. We applied longitudinal data from the China Health and Nutrition Survey (CHNS) and the difference-in-differences (DID) econometric analysis to study this reform’s effect on health insurance coverage rates and the corresponding effect on health service utilisation efficiency.

We report a significant increase in health insurance coverage rates after 2009, showing that the reform indeed provided universal basic insurance coverage to all citizens, including those who were unemployed. However, while the reform increased the overall health insurance coverage rate, it also led to a significant increase in demand for high-quality health care delivery. This was represented by a trend in Chinese citizens, even unemployed individuals, to choose tertiary city hospitals for their health care. We explain this trend as being due to the increased cost coverage by insurance, meaning individuals are more likely to visit upper-level hospitals, since they are now affordable, and more importantly, provide higher-quality medical services. This will harm the second stage goal of the 2009 health care reform, which is to promote the primary health care (PHC) system to increase overall health care system utilisation.

This study is subject to a few limitations. On the one hand, the survey data applied in our analysis give only rough variable estimates. This requires a more comprehensive investigation in the future to access more accurate results, especially in distinguishing among disease types to evaluate the necessity of visiting tertiary hospitals. On the other hand, the most recent survey data, especially from more recent years, are not yet available for investigation. This may result in a lagging review of the 2009 reform in terms of health service utilisation. We will thus continue to evaluate the reform’s effects in future studies.

Nevertheless, this paper’s findings have several implications. Our results imply that China’s 2009 health care reform’s elements under the existing health insurance scheme need to continually allocate health infrastructures. This may in turn strengthen the quality and capacity of PHC services, which will eventually strengthen the health care system’s efficiency overall.

## Figures and Tables

**Table 1:** The take-up status of health insurance by types and waves.

	Overall Coverage	Commercial	Free Insurance	Urban System		Rural System	Others
(%)				UEBMI	URBMI	NRCMS	
Urban							
2006	49.51	8.34	10.77	13.29	NA	20.34	5.04
2009	86.34	5.95	7.06	39.7	17.74	34.03	2.04
2011	92.03	6.77	5.13	45.52	23.9	24.33	1.8
2015	96.09	3.99	4.49	45.47	27.67	25.5	0.82
Rural							
2006	47.63	3.71	3.89	4.61	NA	78.21	0.60
2009	92.61	2.41	2.65	9.32	8.45	78.83	0.57
2011	96.57	3.36	2.78	13.46	11.77	70.35	0.61
2015	97.77	2.02	1.92	14.77	13.51	70.45	0.39

Note: This table presents health insurance usage by urban and rural districts among the four time waves. UEBMI denotes for Urban Employees’ Basic Medical Insurance, URBMI is Urban Resident Basic Medical Insurance, and NRCMS is New Rural Cooperative Medical Scheme. The total sample includes 41,759 individuals (15,120 from urban areas and 26,639 from rural areas). The last column (Others) includes health insurance for women and children.

**Table 2:** Summary of key variable statistics.

Variable	Definition
Insurance coverage	The proportion of respondents that are covered by at least one public health insurance
Health service utilisation	The proportion of individuals who accessed any formal health care in the previous
	4 weeks to individuals who reported to feel sick in the previous 4 weeks
Tertiary hospital choice	The proportion of individuals who visited tertiary hospitals to the number of individuals
	who reported to seek formal medical care in the previous 4 weeks
OOP expenditure	Out-of-pocket expenditure that is not covered by insurance
Unemployed	The proportion of respondents who report not at work relative to the entire sample
Urban	The proportion of respondents who live in a city, town or county capital city
	relative to the entire sample
Age	The age of respondents
Years of education	The years of education of respondents

**Table 3:** Trends in health service utilisation and choice of tertiary hospitals, 2006–2015.

	Before		After	
	*n*	%(95 CI)	*n*	%(95 CI)
Seeking formal medical care when ill				
All	6543	19.70 (15.18–24.22)	9480	32.26 (28.07–36.45)
Urban	2342	22.22 (15.24–29.21)	5388	34.00 (28.01–39.98)
Rural	4201	17.36 (11.52–23.19)	4092	30.03 (24.23–35.82)
Tertiary Hospital Choice				
All	1018	59.55 (49.16–69.95)	1742	67.13 (61.37–73.02)
Urban	348	59.18 (45.09–73.28)	823	67.76 (60.27–75.25)
Rural	670	60.00 (44.41–75.59)	919	66.34 (57.03–75.64)

**Table 4:** Summary statistics of key variables.

Variable	Full Sample		2006		2009		2011		2015	
	Mean	Std	Mean	Std	Mean	Std	Mean	Std	Mean	Std
Insurance coverage (%)	0.849	0.359	0.490	0.500	0.906	0.291	0.948	0.221	0.971	0.168
OOP Expenditure (Yuan)	958	4537	699	2854	855	3313	821	4374	1284	5840
Female (%)	0.526	0.499	0.524	0.499	0.520	0.500	0.529	0.499	0.529	0.499
Unemployed (%)	0.052	0.221	0.017	0.130	0.017	0.128	0.009	0.094	0.009	0.096
Urban (%)	0.377	0.485	0.343	0.475	0.340	0.474	0.411	0.492	0.395	0.489
Age (Years)	56.812	15.913	57.749	16.196	57.083	16.168	56.774	15.816	55.942	15.551
Years of education	8.212	2.391	8.342	2.580	8.143	2.229	8.156	2.438	8.174	2.261
Observations	45,627		9661		9987		13,035		12,944	

Note: This table summarises the statistics of the key variables among the four time waves. The total sample includes 45,627 individuals.

**Table 5:** Treatment effect of health care reform on insurance coverage.

	(1)	(2)	(3)
Variable	General Result	Controlled DID	Triple DID
After 2009	0.333 ***	0.339 ***	0.408 ***
	(0.0234)	(0.0236)	(0.0384)
Unemployed	−0.333 ***	−0.326 ***	−0.204 ***
	(0.0321)	(0.0328)	(0.0432)
Unemployed * postreform	0.310 ***	0.301 ***	−0.307 ***
	(0.0473)	(0.0475)	(0.0672)
Unemployed * urban			0.183 ***
			(0.0623)
Urban * postreform			−0.121 **
			(0.0483)
Unemployed * postreform * urban			0.278 ***
			(0.100)
Urban			0.102 ***
			(0.0375)
Age		0.00143 *	
		(0.000727)	
Gender		0.0217	
		(0.0204)	
Constant	0.638 ***	0.552 ***	0.576 ***
	(0.0184)	(0.0442)	(0.0293)
Observations	1175	1175	1175
R-squared	0.334	0.337	0.347

Note: This table presents the treatment effect of the new health care reform by using the difference-in-differences method. Standard errors are presented in parentheses: *** denotes *p* < 0.01, ** denotes *p* < 0.05, and * represents *p* < 0.1.

**Table 6:** The estimation of the 2009 health care reform’s effect on people’s decisions to visit tertiary hospitals.

Variable	Coefficient	Odds Ratio	95% CI
% Cost covered	0.03	1.02	[1.01, 1.03]
	(0.00) ***	(0.00) ***	
OOP	0.85	1.00	[0.99, 1.00]
	(0.07) *	(0.07) *	
% Cost covered * OOP	−0.01	0.99	[0.99, 1.00]
	(0.06) *	(0.06) *	
Age	−0.01	1.01	[0.99, 1.03]
	(0.34)	(0.34)	
Urban	2.86	0.98	[0.54, 1.81]
	(0.97)	(0.97)	
Unemployment	−1.31	0.66	[0.17, 2.61]
	(0.55)	(0.55)	
Female	0.04	0.97	[0.52, 1.83]
	(0.93)	(0.93)	
Pseudo R${}^{2}$	0.12		

Note: This table presents the logistic estimation of the 2009 health care reform’s effect on people’s decisions to visit tertiary hospitals. *p* values are presented in parentheses; *** denotes *p* < 0.01, ** denotes *p* < 0.05, and * denotes *p* < 0.1.

**Table 7:** Robustness check: treatment effect of health care reform on health care utilisation.

	(1)	(2)
Variable	General Result	Controlled DID
After 2009	0.0030	0.0035
	(0.0027)	(0.0027)
Urban	0.0357 ***	0.0347 ***
	(0.0034)	(0.0034)
Urban * postreform	0.0148 ***	0.0154 ***
	(0.0045)	(0.0045)
Age		0.0007 ***
		(0.0000)
Gender		0.0071 ***
		(0.0021)
Constant	0.0080 ***	−0.0372 ***
	(0.0020)	(0.0044)
Observations	22,697	22,697
R-squared	0.019	0.025

Note: This table presents the robustness check of the 2009 health care reform’s effect on health care utilisation. Standard errors are presented in parentheses, and *** denotes *p* < 0.01, ** denotes *p* < 0.05, and * denotes *p* < 0.1.

**Table 8:** Robustness check: treatment effect of health care reform on insurance coverage.

	(1)	(2)	(3)
Variable	General Result	Controlled DID	Triple DID
After 2009	0.364 ***	0.366 ***	0.294 ***
	(0.0584)	(0.0578)	(0.101)
Unemployed	−0.184 ***	−0.227 ***	−0.293 ***
	(0.0509)	(0.0504)	(0.0901)
Unemployed * postreform	0.118 **	0.113 *	0.189 *
	(0.0590)	(0.0584)	(0.109)
Unemployed * urban			0.242 **
			(0.102)
Urban * postreform			0.101
			(0.124)
Unemployed * postreform * urban			−0.221 *
			(0.125)
Urban			−0.111
			(0.108)
age		0.00278 ***	
		(0.000198)	
female		−0.00596	
		(0.00786)	
Constant	0.630 ***	0.512 ***	0.706 ***
	(0.0505)	(0.0509)	(0.0897)
Observations	9208	9208	9208
R-squared	0.288	0.303	0.292

Note: This table presents the 2009 health care reform’s treatment effect on insurance coverage by using the DID method. Standard errors are presented in parentheses, and *** denotes *p* < 0.01, ** denotes *p* < 0.05, and * denotes *p* < 0.1.

## Abbreviations

The following abbreviations are used in this manuscript:

PHC	Primary health care
LIP	Labour Insurance Programme
SOE	State-owned enterprise
GIP	Government insurance programme
UEBMI	Urban Employees’ Basic Medical Insurance
URBMI	Urban Resident Basic Medical Insurance
NRCMS	New Rural Cooperative Medical Scheme
CHNS	China Health and Nutrition Survey
DID	Difference-in-differences
